# Bullous Pemphigoid: A Spontaneous Presentation in a Patient With Chronic Kidney Disease

**DOI:** 10.7759/cureus.25806

**Published:** 2022-06-10

**Authors:** Brett Brazen, Ariel Kidron, Zakaria Sheikh, Vikeerna Kamatgi, Sann Htoo

**Affiliations:** 1 Dermatology, Aventura Hospital and Medical Center, Aventura, USA; 2 Emergency Medicine, Nova Southeastern University Kiran C. Patel College of Osteopathic Medicine, Fort Lauderdale, USA; 3 Internal Medicine, Aventura Hospital and Medical Center, Aventura, USA

**Keywords:** ckd(chronic kidney disease), autoimmune bullous dermatosis, clinical dermatology, pemphigoid, bullous dermatoses

## Abstract

Bullous pemphigoid (BP) is an autoimmune subepidermal blistering pathology characterized by the development of pruritic, tense bullae and blisters on the lower extremities, axilla, and trunk. Its dermatopathology entails autoantibodies that target hemidesmosomes located in the basement membrane. The disease typically manifests in individuals over 50 years old with a higher prevalence in patients with concurrent neurological or dermatological autoimmune diseases. In this report, we discuss a case of a 67-year-old male who presented with a one-month history of itchy blisters occurring bilaterally in the lower extremities. The manifestation of BP, its pathophysiology, and treatment modalities are explored, We also engage in a review of the relevant literature.

## Introduction

Bullous pemphigoid (BP) is a rare autoimmune skin disease that causes large, diffusely eczematous, pruritic fluid-filled blisters often in areas that flex, such as the lower trunk, axilla, and upper thighs. Our case was unique in that the bullous lesions occurred on the extensor areas in the lower extremities. The disease commonly occurs in the elderly population with more severity manifested in individuals over 50 years old with concurrent renal disease [1-3]. The dermatopathology highlights IgG antibodies that act against the structural aspects of keratinocytic hemidesmosomal proteins BP180 and BP230 [4]. In the United States, the overall prevalence of BP has been reported to be 0.012% per 100,000 individuals with incremental increases within each successive age group over the age of 60 years [5]. The diagnosis of BP is established using clinical, histological, and immunological modalities with the first-line treatment being anti-inflammatory immunosuppressive pharmacology directed at reducing the rogue antibodies. In addition, due to the rarity of BP and the similarities that lesions share with other dermatological disorders such as epidermolysis bullosa acquisita, porphyria cutanea tarda, and bullous contact dermatitis, the condition is often misdiagnosed, and proper treatment is delayed leading to poor patient outcomes [6]. This case serves to remind clinicians of all specialties about the classical presentation of BP, available treatment therapies, and the importance of a thorough dermatological examination.

## Case presentation

**Figure 1:**
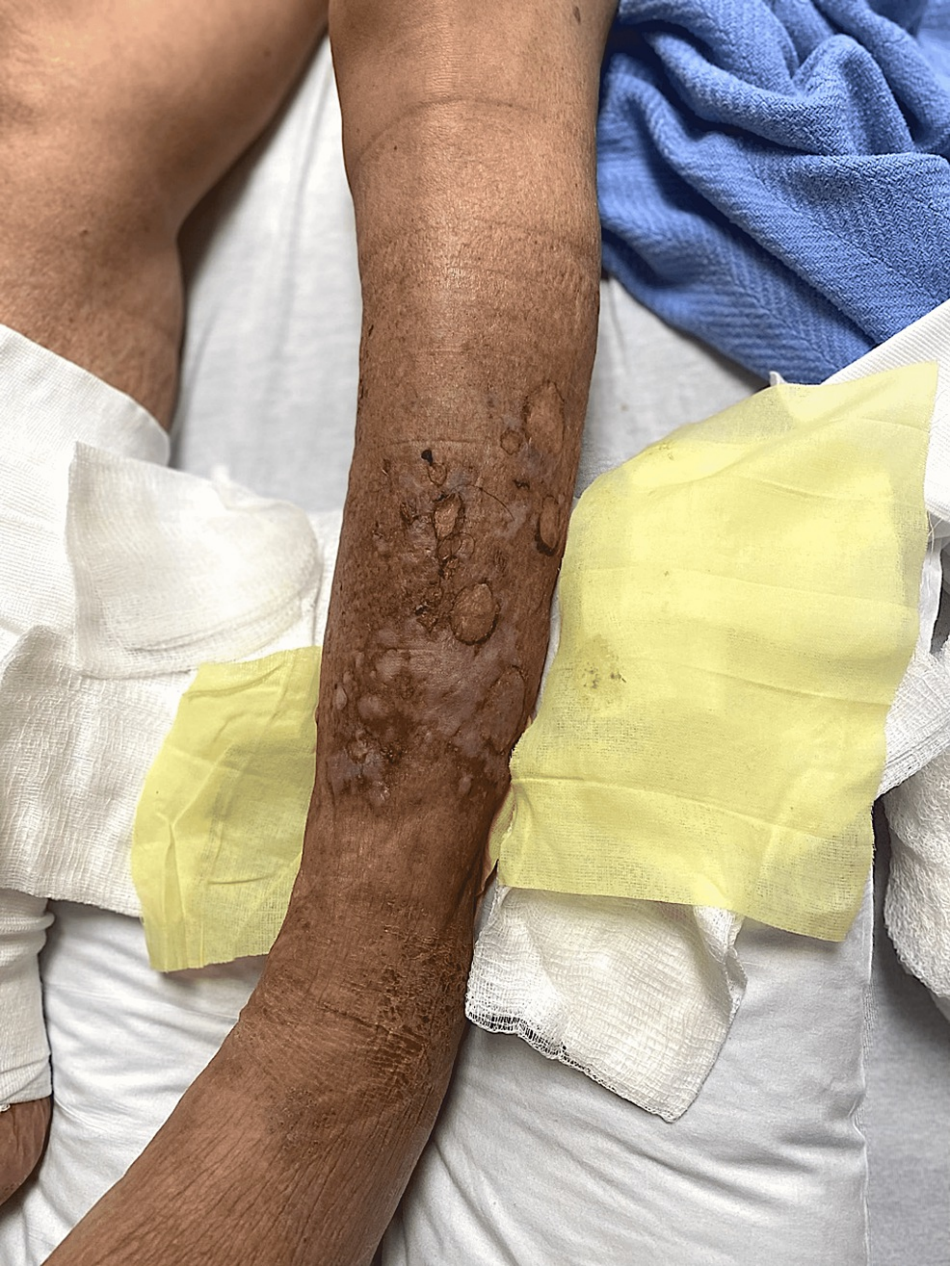
Ruptured bullae on the anterior aspect of the left shin with visible subcutaneous fat

**Figure 2:**
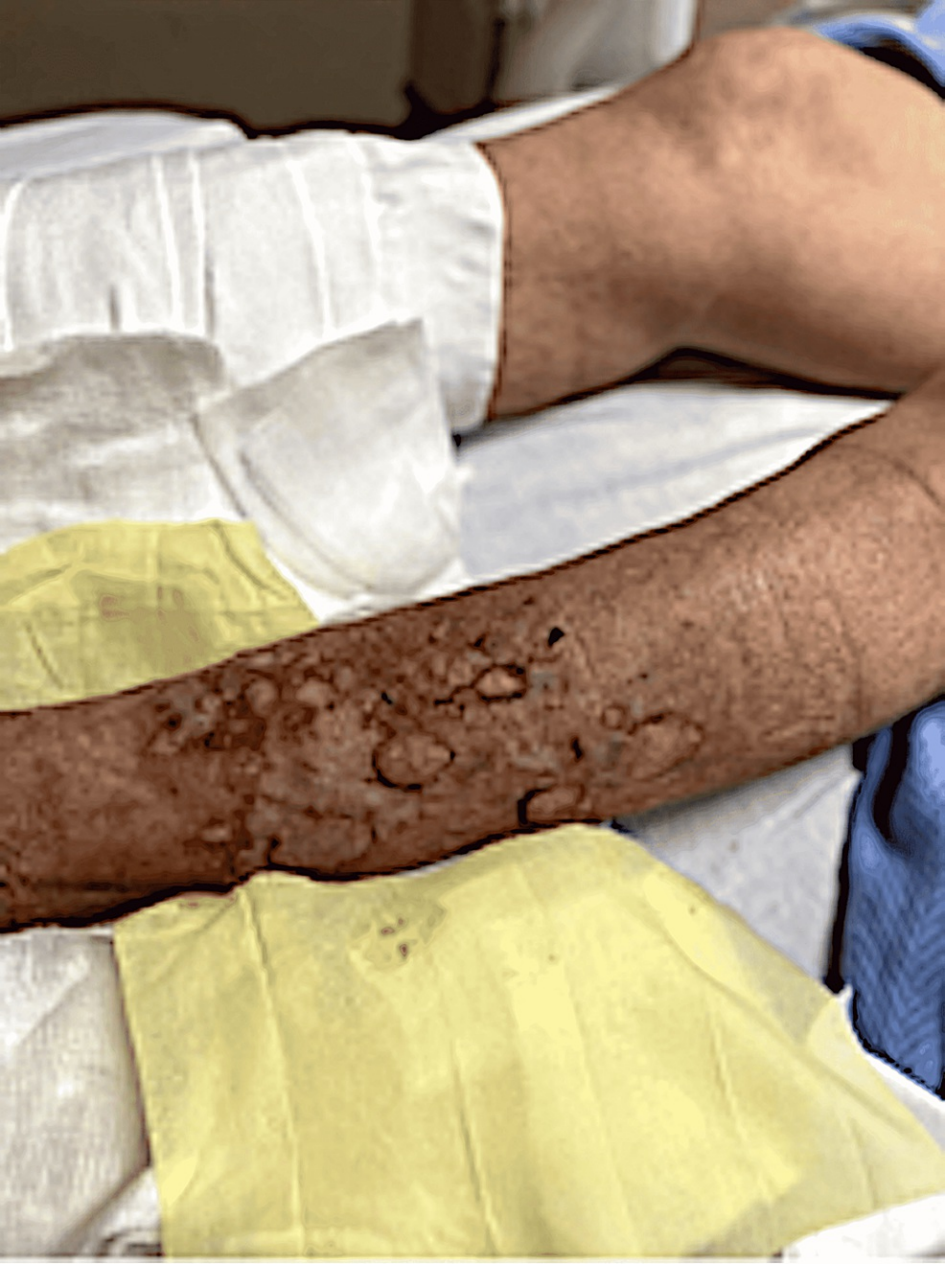
Lateral view of ruptured bullae on the anterolateral aspect of the left shin with visible subcutaneous fat

**Figure 3:**
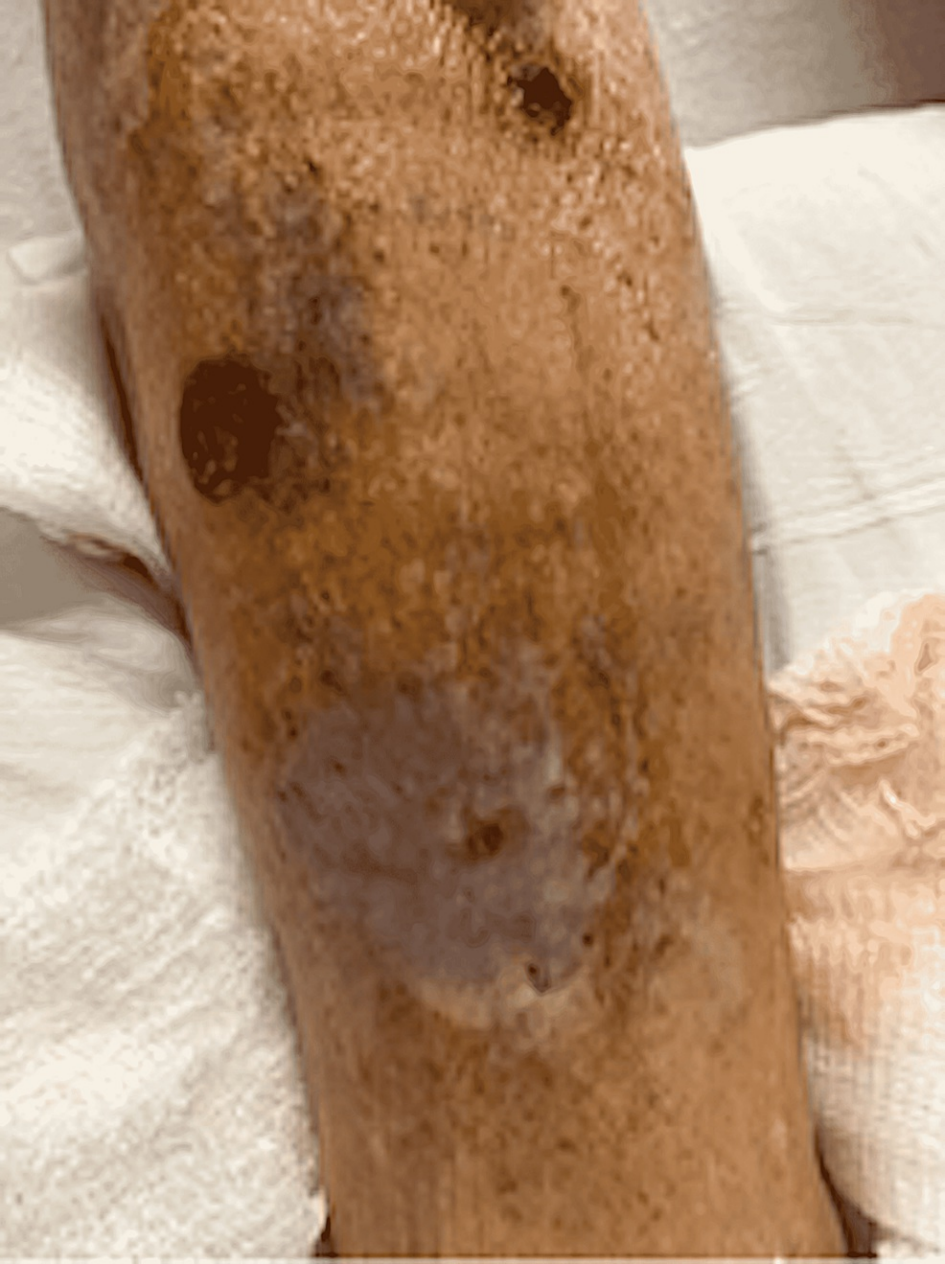
Ruptured bullae on the anterolateral aspect of the right shin in various stages of healing. Lichenified skin on the distal lower extremity can be noted

A 67-year-old male with a previous medical history of atrial fibrillation, stroke, and chronic kidney disease (CKD) stage III presented to the emergency room for the evaluation of worsening creatinine clearance and bullous blistering lesions present over the bilateral lower extremities for a duration of one month (Figures 1, 2). Initially, the patient reported the lesions were warm and erythematous, without bullae, covering the anterior portions of the bilateral lower extremities. The patient denied excessive sun exposure, using new detergents, or extensive contact with foliage. During his hospitalization, the patient’s bullae were wrapped daily in Xeroform dry sterile dressings. The patient had been seen by his primary care provider and prescribed a 10-day course of ciprofloxacin without any significant improvement. He had been further evaluated by his outpatient dermatologist who had obtained a punch biopsy, the findings of which had shown subepidermal blistering consistent with BP. He had been subsequently prescribed clobetasol 0.05% ointment. During the course of the treatment, the patient developed the tense bullae described above and subsequently presented to the emergency room for further evaluation. The patient endorsed a similar blistering episode a couple of years prior, which had resolved with the administration of clobetasol 0.05% ointment and silver sulfadiazine 1% ointment. After three days of treatment, the lesions showed significant healing with a reduction in the size of the bullae, and resolution of the pruritis, but with residual erythema (Figure 3). The patient was instructed to follow up with his dermatologist for continuity of treatment for the skin lesions with steroids.

## Discussion

The literature recommends the use of the Bullous Pemphigoid Disease Area Index (BPDAI) as an objective clinical assessment measure of disease severity using measures of skin and mucosal involvement with two activity components, one for the extent of blistering, and one for the extent of urticarial/eczematous change. Scores totaling <19 correspond to mild disease severity, >20 and <56 correspond to moderate disease, and those >57 are labeled as severe. The BPDAI has proven to directly correlate with antibody titers and has shown significant inter-rater reliability [7]. The clinical diagnosis of BP may be challenging as the skin lesions are similar to an array of other blistering disorders, most commonly pemphigus vulgaris (PV). One French study found that the presence of three out of four clinical criteria, including the absence of atrophic scars, limited head or neck involvement, absence of mucosal involvement, and age greater than 70 years, was associated with a sensitivity of 90%, specificity of 83%, and a positive predictive value of 95% in diagnosing BP [8]. In addition, serodiagnosis has been shown to exhibit high sensitivity and specificity in distinguishing between the pathologies. According to the literature, there is a positive incidence correlation between immune disorders involving the two basement membranes belonging to the skin and kidney with the severity of the BP skin lesions often paralleling that of the kidney disease. Moreover, the development of BP has been cited in certain cases to occur secondary to kidney diseases such as membranous nephropathy [3]. Furthermore, CKD may predispose patients to renal infections, which may trigger a secondary BP flair given the activation of the immune system [9].

Commonly, tissue-bound and circulation autoantibodies targeting BP180 and BP230 have been isolated in patients with BP and correlate with disease severity. Eosinophilic tissue infiltration serves as functional antigen-presenting cells with direct cleavage of BP180. This distinguishes BP when compared to anti-Dsg1 and anti-Dsg3 antibodies present in PV [10-14]. Ultimately, the gold standard for diagnosis of BP is direct immunofluorescence of a skin biopsy from the perilesional tissue with characteristic linear IgG and C3 deposits along the basement membrane [15]. The subepidermal blistering seen in BP aids in distinguishing the disease from PV, which shows supra basilar splitting with IgG and C3 deposition in a smooth and reticular pattern on an epithelial layer, referred to as “fishnet-like,” or epidermolysis bullosa acquisita (EBA), which shows moderate-to-thick linear IgA, IgG, and C3 deposition along the dermal-epidermal junction (DEJ). Clinically, the lesions seen in BP usually lack the oral mucosal involvement and positive Nikolsky sign seen in PV, nor the skin fragility and development of blisters overlying areas with repeated minor trauma such as the extensor surfaces, hands, and feet seen in EBA [16-18].

The mainstay of treatment for BP in the setting of kidney disease consists of both topical and systemic steroids depending on the severity of manifestations. Classified into types ranging from mild to severe, patients with localized, mild-to-moderate disease manifestations can experience adequate resolution from treatment with topical corticosteroids alone. For those with diffuse or severe manifestations, oral corticosteroids, generally prednisone, are utilized either in conjunction with topicals or as a monotherapy. Adjunctive therapies have also been described, notably doxycycline, mycophenolate mofetil, and azathioprine [18,19]. Recent studies have investigated and shown promising results using monoclonal antibodies such as rituximab, omalizumab, and reslizumab as an alternative therapy with a better safety profile in patients resistant to high-dose steroids or traditional immunosuppressive therapy. Omalizumab has shown exciting potential with a recent study finding complete response rates as high as 80% and a mean recurrence time of 3.4 months. These biologics with mechanisms of action including inhibition of CD-20 proteins on B-lymphocytes, downregulation of IgE cell surface receptors on eosinophils, and IL-5 inhibition have shown great potential for future research into steroid-sparing treatments of BP [14,19].

## Conclusions

BP is the most common autoimmune blistering disorder. Especially prevalent in the elderly population, it presents with large, diffusely eczematous, pruritic fluid-filled blisters, usually on the lower trunk, axilla, and upper thighs. BP is diagnosed clinically and confirmed by biopsy and immunofluorescence. The lesions are typically confined to non-mucosal surfaces and demonstrate a negative Nikolsky sign. The gold standard for diagnosis is direct immunofluorescence with characteristic linear IgG and C3 deposits along the basement membrane. Conventional treatment regimens include topical steroids for localized or mild-to-moderate cases, while severe manifestations are treated with oral corticosteroids. Alternative therapies have been utilized for patients resistant to steroids and further research is being conducted to explore the future potential of biologic medications in an adjunctive role. Given the severity and challenging presentation of the disease, we hope our report provides awareness about the typical presentation of BP and can serve as a guideline for proper diagnostic and treatment regimens.

## References

[1] Ngan V (2022). Pemphigus vulgaris. DermNet NZ.

[2] Ikeda M, Honda H, Kobayashi N (2017). Membranous glomerulonephropathy in a patient with bullous pemphigoid. CEN Case Rep.

[3] Ross EA, Ahmed AR (1989). Bullous pemphigoid-associated nephropathy: report of two cases and review of the literature. Am J Kidney Dis.

[4] Parellada J, Olivera Arencibia Y, Watson H, Parellada N, Saikaly LE, Saikaly SK (2018). A case of bullous pemphigoid: a prevalent and potentially fatal condition. Cureus.

[5] Wertenteil S, Garg A, Strunk A, Alloo A (2019). Prevalence estimates for pemphigoid in the United States: a sex-adjusted and age-adjusted population analysis. J Am Acad Dermatol.

[6] Joly P, Benichou J, Lok C (2005). Prediction of survival for patients with bullous pemphigoid: a prospective study. Arch Dermatol.

[7] Cole EF, DeGrazia T, Sun Y, Liu Y, Feldman RJ (2021). Assessing disease outcome measures in bullous pemphigoid on standard-of-care therapies. JID Innov.

[8] Vaillant L, Bernard P, Joly P (1998). Evaluation of clinical criteria for diagnosis of bullous pemphigoid. French Bullous Study Group. Arch Dermatol.

[9] Moro F, Fania L, Sinagra JL, Salemme A, Di Zenzo G (2020). Bullous pemphigoid: trigger and predisposing factors. Biomolecules.

[10] Thoma-Uszynski S, Uter W, Schwietzke S (2004). BP230- and BP180-specific auto-antibodies in bullous pemphigoid. J Invest Dermatol.

[11] Sitaru C, Dähnrich C, Probst C (2007). Enzyme-linked immunosorbent assay using multimers of the 16th non-collagenous domain of the BP180 antigen for sensitive and specific detection of pemphigoid autoantibodies. Exp Dermatol.

[12] Feng S, Lin L, Jin P, Wu Q, Zhou W, Sang H, Shao C (2008). Role of BP180NC16a-enzyme-linked immunosorbent assay (ELISA) in the diagnosis of bullous pemphigoid in China. Int J Dermatol.

[13] Schmidt E, Obe K, Bröcker EB, Zillikens D (2000). Serum levels of autoantibodies to BP180 correlate with disease activity in patients with bullous pemphigoid. Arch Dermatol.

[14] Rhyou HI, Han SH, Nam YH (2021). Successful induction treatment of bullous pemphigoid using reslizumab: a case report. Allergy Asthma Clin Immunol.

[15] Fuertes de Vega I, Iranzo-Fernández P, Mascaró-Galy JM (2014). Bullous pemphigoid: clinical practice guidelines. Actas Dermosifiliogr.

[16] Arpita R, Monica A, Venkatesh N, Atul S, Varun M (2015). Oral pemphigus vulgaris: case report. Ethiop J Health Sci.

[17] Kumetz EA, Meyerle JH, Rivard SC (2020). Epidermolysis bullosa acquisita: a case report. Am J Case Rep.

[18] Di Lernia V, Casanova DM, Goldust M, Ricci C (2020). Pemphigus vulgaris and bullous pemphigoid: update on diagnosis and treatment. Dermatol Pract Concept.

[19] Joly P, Roujeau JC, Benichou J (2002). A comparison of oral and topical corticosteroids in patients with bullous pemphigoid. N Engl J Med.

